# Ventricular Arrhythmia Discriminator Programming and the Impact on the Incidence of Inappropriate Therapy in Patients with Implantable Cardiac Defibrillators

**Published:** 2007-04-01

**Authors:** Uma N Srivatsa, Bobbi L Hoppe, Sanjiv Narayan, Gregory K Feld, Ulrika Birgersdotter-Green

**Affiliations:** UCSD Medical Center, and the Veterans Administration Medical Center, San Diego, CA.

**Keywords:** Implantable Cardiac Defibrillators, Inappropriate Therapy, Ventricular Arrhythmia Discriminators

## Abstract

**Background:**

The incidence of inappropriate therapy from implantable cardioverter defibrillators (ICDs) has been reduced by programming ventricular arrhythmia discriminators (VAD) on at the time of implant.

**Objective:**

To determine which VAD is most effective in preventing inappropriate therapy.

**Methods and Results:**

Dual chamber ICD (n=48) or cardiac resynchronization therapy defibrillator (CRT-D) (n=55) implantation was performed in 103 patients (M=94, F=9). Patients were followed prospectively for therapy events (shock or anti-tachycardia pacing) for a mean 362±289 days.  Events were correlated with clinical characteristics and VAD programming.  Of the 103 pts followed, 11 received inappropriate therapy (IT), 15 received appropriate therapy (AT), and 77 received no therapy (NT). In the AT and IT groups, a total of 207 events (ATP=171, shock=36) were observed. A total of sixty-four electrograms (EGMs) were analyzed.  Programming VADs "ON" versus "OFF" reduced the incidence of IT events compared to those receiving AT or NT events (p<.01), with a trend in fewer patients receiving IT (31.3% "ON" vs 55.6% "OFF", p = 0.131).  Programming atrial fibrillation (AF) detection ON resulted in fewer patients receiving IT compared to those receiving AT or NT (3.6% vs 19%, p<.05). Furthermore, programming AF or AFL algorithms "ON", resulted in overall fewer episodes of IT therapy (p<.01).

**Conclusion:**

AF or AFL discriminators significantly reduced the incidence of IT, and were predominantly responsible for the benefits from VAD programming observed in this study.  Activating these features as part of routine ICD or CRT-D programming may provide a simple and effective alternative to the use of more complex and multiple VAD strategies.

## Background

The incidence of inappropriate therapy in patients with implantable cardioverter defibrillators (ICDs) has been well documented [1,2]. The incidence ranges from 15-40% and often occurs within the first 6 months of follow-up [2]. Inappropriate shocks have been correlated with a decrease in the quality-of-life (QOL) and may have negative psychological consequences [3].

The high incidence of inappropriate therapy has led to the development of ventricular arrhythmia discriminators (VADs). Although VAD programming has resulted in a reduced inappropriate therapy (IT) in patients with single chamber or dual chamber ICDs [4], the benefit of VAD programming in patients receiving cardiac resynchronization therapy defibrillator (CRT-D) remains unknown.

The benefit of VAD programming in patients with CRT-Ds may be more pronounced, since the incidence of IT in patients with CRT-D was quite high (11%) in the MIRACLE-ICD trial [5]. In addition, the rare pro-arrhythmic effect of biventricular pacing has also been documented in select patients with CRT-D devices [6] and may lead to increased ventricular arrhythmia burden in these patients.

VAD programming is often at the discretion of the implanting physician and some physicians enable discriminators only after inappropriate therapy has been delivered [7].  Though implanting physicians have a choice of which of the VADs algorithms to be programmed on, it remains unclear which, if any, single discriminator is more effective in preventing IT.

We hypothesized that programming VAD on at the time of CRT-D implantation would decrease the number of IT events. In addition, we set forth to determine whether a particular VAD would be more effective in preventing IT. These results would have important implications for routine programming of both ICD and CRT-D therapy.

## Methods

This study was approved by the joint University of California/Veterans Administration Medical Center, San Diego, Institutional Review Board for human investigation. Consecutive patients who underwent clinically approved dual chamber ICD or CRT-D implantation between January 2001 and April 2004 were followed prospectively. Dual chamber ICD implantation consisted of insertion of an atrial bipolar electrode and a right ventricular bipolar electrode. CRT-D device configuration at implantation consisted of a left ventricular unipolar electrode combined with a bipolar right ventricular pace/sense electrode connected with a "Y" adapter to a single ICD ventricular port or to a separate left and right ventricular ports and an atrial bipolar electrode. VADs were programmed individually according to clinical characteristics and physician preference (Table 1).  Baseline patient clinical data, 12 lead ECG and implant data and settings were recorded.

## Statistical Analysis

Continuous variables are reported as the mean ± standard deviation.  Dichotomous variables are reported as percentages and were compared using the chi-square and fisher exact test.  A p value =.05 was considered statistically significant.  Logistic regression was used to predict the power of different variables in a multivariate analysis.

## Follow-up

Data obtained during routine ICD/CRT-D interrogation during regularly scheduled follow-up visits at 3, 6, 12, and 18 months, were analyzed with respect to appropriate vs. inappropriate arrhythmia discrimination and therapy. An inappropriate therapy event was defined as ATP or shock delivered due to rhythms that were incorrectly characterized by the device. Each event was considered an independent event even if it occurred in the same patient. All therapy events documented by intracardiac electrograms (EGMs) were evaluated independently by two electrophysiologists and classified as inappropriate or appropriate based on data retrieved from the CRT-D at the time of routine follow-up. The index arrhythmia, therapy delivered, and resultant rhythm were recorded, and any device reprogramming, including VAD programming, performed during follow-up was noted.

## Results

### Patient Characteristics

One hundred and seven patients who received ICD or CRT-D with appropriate follow up in our institution were identified. Three patients receiving devices without an atrial lead and one patient who received both inappropriate and appropriate therapy were excluded from analysis. Baseline characteristics for the 103 patients undergoing ICD/CRT-D implantation included in the study are listed in Table 2, including those in patients who received AT (n=15) and IT (n=11). There were no statistically significant differences in baseline characteristics, including medication usage. Programmable VAD algorithms included in this analysis are listed in Table 2. VADs were programmed "ON" in 78/103 (75.7%) patients at the time of implant.

### Follow-up Period

During a mean follow-up of 362 ± 289 days, 15 (14.5%) patients received appropriate therapy, 11 (10.7%) patients received inappropriate therapy (Table 3), and 77 (74.8%) patients received no therapy. Of the 26 patients receiving therapy, a total of 207 therapy events (ATP=171, shock=36) were observed. Of these 207 therapy events, 64 available EGMS were analyzed by two independent electrophysiologists. Forty (62.5%) events were appropriate, whereas 24 (37.5%) were inappropriate. Inappropriate therapy events were significantly more common (χ^2^; p=0.01) when VAD was turned off (5/24, 20.8%) versus on (6/79, 7.6%).

### Individual Ventricular Arrhythmia Discriminators

Programming atrial fibrillation (AF) ON versus OFF resulted in significantly fewer patients receiving IT compared to those receiving AT or NT (3.6% vs 19%, p<.05). Furthermore, among individual ATP or shock events analyzed by EGM, programming AF or AFL "ON" (p<0.001; for both) reduced IT events. Onset, stability, V>A, sinus tachycardia, and algorithms did not show additional benefit in reducing inappropriate therapy. We did not analyze the morphology criteria as there were very few patients with this feature available at the time of the study.

### Predictors of Inappropriate vs. Appropriate Therapy

Baseline clinical differences between patients receiving any (not shown), inappropriate, or appropriate therapy (Table 2) were not statistically different (p=NS).

### Safety

In this analysis, none of the patients who had VADs turned on had ventricular arrhythmias that went untreated.

## Discussion

This study showed that programming at least one VAD at the time of implant greatly reduced the incidence of inappropriate therapy events in patients receiving dual chamber ICDs or CRT-D. Despite the potential benefit of VAD programming, only 75.7% of patients had a VAD programmed on at the time of implant. This has important clinical implications as CRT-D device implantation is becoming more widespread and more cardiologists are implanting CRT-D and routinely following patients post-implant.

Programming VADs "ON" is safe and has not resulted in failure of detection of ventricular arrhythmias [10]. Therefore, given the potential benefit, we advocate that ICD/CRT-D implanting physicians should routinely consider programming VADs on at the time of implant. In addition, special consideration should be given to those patients with a history of underlying atrial arrhythmias, since these patients are more likely to receive inappropriate therapy [11].

**Figure 1:**
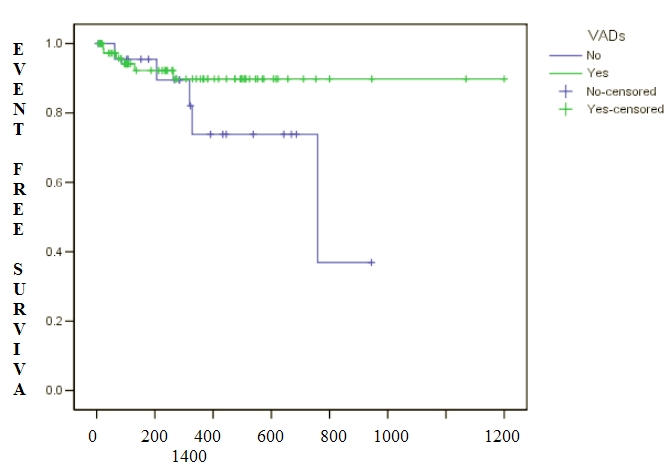
Kaplan Meier analysis of time (days) until first inappropriate therapy (shock/anti-tachycardia pacing in patients with any VAD programmed "ON" compared to those without any VAD programmed "ON".

Interestingly, there also appears to be a time dependent relationship between programming a VAD "ON" until first inappropriate therapy delivered as represented in Figure 1. This benefit appears to occur within the first year of implant and suggests that VAD programming "ON" provides protection from first IT.

Of the individual VADs available, AF and AFL discriminators significantly reduced IT events in this patient population (p<0.001 for each). It is plausible that the relatively high incidence of atrial arrthyhmias (26.2%) in this patient population, accounts for this finding, however, this is reflective of atrial arrhythmias found in the similar patient populations.

Individual discriminators differ between device manufacturers and are often comprised of multiple algorithms based on the atrial rate versus ventricular rate, the occurrence of P waves at different locations of the R-R interval, and P/R association. Unfortunately, our sample size precluded a comparison of AF discrimination algorithms between manufacturers.

Previous studies have investigated whether clinical variables predict the delivery of appropriate versus inappropriate therapy in patients with ICDs. Nanthakumar, et.al. observed that patients with AF or NYHA Class I failure were at higher risk of inappropriate shocks [11].  More recently, in a study comprised of children and adolescents, Korte et. al, did not identify any clinical variable that predicted either appropriate or inappropriate therapy [12].  In the present study, clinical baseline characteristics did not predict those patients receiving any therapy, whether appropriate or not.

## Conclusions

Programming VAD "ON" is safe after device implantation and results in reduced incidence of inappropriate therapy in patients receiving both prophylactic ICDs and those receiving CRT-D devices.  In terms of traditional individual discriminators, atrial fibrillation and atrial flutter algorithms may be the single most effective VAD for reducing inappropriate therapy given the high incidence of atrial arrhythmias in this population. However, newer algorithms incorporating QRS morphology warrant further investigation.

## Limitations

This a small study consisting of a predominantly male tertiary referral population and may not reflect a larger community population. Since this study was a prospective observational analysis, randomization to activation or inactivation of VAD was not performed, but should form the basis for a prospective randomized study to evaluate the optimal VAD in different patient populations. Furthermore, even though only a limited number of events are typically stored in any device; this analyzed sample is likely to be a fair representation of the entire burden of events. Lastly, discriminators including QRS morphology could not be fully evaluated since they were available in only a few patients in our study population.

## Figures and Tables

**Table 1:**
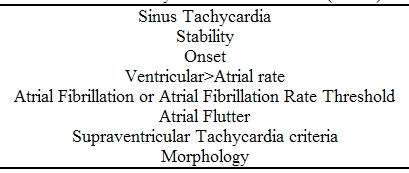
Examples of Ventricular Arrhythmia Discriminators (VADs)

**Table 2:**
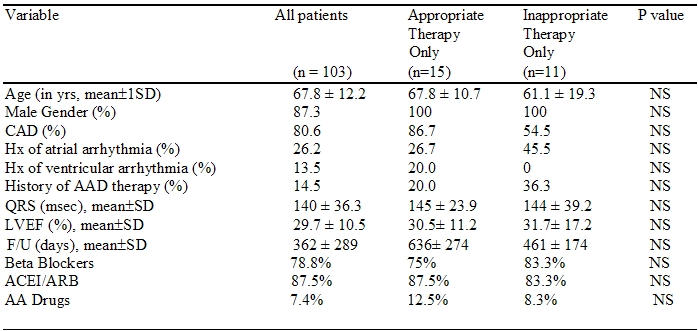
Patient Characteristics

ACEI/ARB = angiotensin converting enzyme inhibitor/angiotensin receptor blocker, AA drugs  = antiarrhythmic drugs,  CAD = coronary artery disease, F/U = followup, Hx = history, LVEF = left ventricular ejection fraction

**Table 3:**
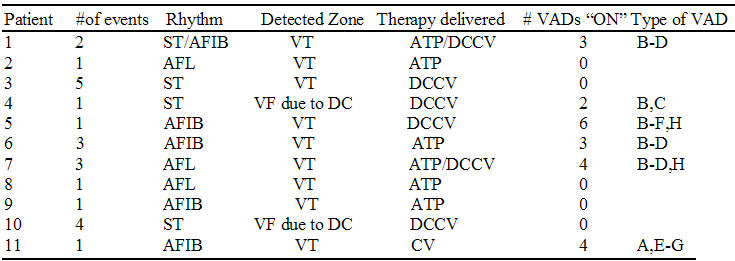
Inappropriate Therapy Events

ST= sinus tachycardia; AFIB= atrial fibrillation; AFL= atrial flutter; VT= ventricular tachycardia; VF= ventricular fibrillation; DC= double counting; ATP= anti-tachycardia pacing; DCCV= direct current cardioversion; VADs= ventricular arrhythmia discriminators;  A =  Sinus Tachycardia, B = Stability, C = Onset, D = Ventricular>Atrial rate, E = Atrial Fibrillation or Atrial Fibrillation Rate Threshold, F = Atrial Flutter, G =  Supraventricular Tachycardia criteria, H =  Morphology
